# Safety and efficacy of obinutuzumab in Chinese patients with B-cell lymphomas: a secondary analysis of the GERSHWIN trial

**DOI:** 10.1186/s40880-018-0300-5

**Published:** 2018-05-30

**Authors:** Yan Qin, Yuqin Song, Zhixiang Shen, Xin Du, Wei Ji, Wanling Hsu, Jun Zhu, Yuankai Shi

**Affiliations:** 1 Department of Medical Oncology, Beijing Key Laboratory of Clinical Study on Anticancer Molecular Targeted Drugs, National Cancer Center/Cancer Hospital, Chinese Academy of Medical Sciences and Peking Union Medical College, Beijing, 100021 P. R. China; 2 Department of Lymphoma, Beijing Cancer Hospital, Peking University, Beijing, 100142 P. R. China; 3 Department of Hematology, Ruijin Hospital, Shanghai Jiaotong University, Shanghai, 200025 P. R. China; 4 Department of Hematology, Guangdong General Hospital/Guangdong Academy of Medical Sciences, Guangzhou, 510030 Guangdong P. R. China; 5 Clinical Science, Shanghai Roche Pharmaceuticals Ltd, Shanghai, 201203 P. R. China; 6 Statistics, Roche (China) Holding Ltd, Shanghai, 201203 P. R. China

**Keywords:** Obinutuzumab, B-cell lymphoma, Chronic lymphocytic leukemia, Chinese patients

## Abstract

**Background:**

Patients with relapsed/refractory B-cell lymphomas have limited treatment options. GERSHWIN is an open-label, single-arm, phase Ib study of obinutuzumab monotherapy in Chinese patients with histologically documented CD20^+^ relapsed/refractory chronic lymphocytic leukemia (CLL), diffuse large B-cell lymphoma (DLBCL), or follicular lymphoma (FL). The primary outcome measure of pharmacokinetics has been previously reported. We now present data on the secondary endpoint measures (e.g., safety, and efficacy and pharmacodynamics).

**Methods:**

Patients received 1000 mg obinutuzumab intravenously on days 1, 8, and 15 of cycle 1 (CLL patients; first dose split over 2 days), and on day 1 of cycles 2–8. Each cycle lasted for 21 days; the treatment period was 24 weeks. All subjects receiving at least one dose of obinutuzumab were included in the analysis of safety, efficacy, as well as pharmacodynamics.

**Results:**

A total of 48 patients (> 18 years of age) were enrolled (CLL: 12; DLBCL: 23; FL: 13). The subjects received a median of two lines of anticancer treatment prior to the enrollment. Thirty-five patients (72.9%) had at least one adverse event (AE). The most frequent AE was infusion-related reactions (15 patients; 31.3%), followed by pyrexia (11 patients; 22.9%). Treatment-related AEs were reported in 28 patients (58.3%), and included one death (interstitial lung disease). End-of-treatment (EoT) response rate was 33.3%. Best overall response rate was 47.9%. Most CLL patients achieved a partial response at EoT (58.3%). CD19^+^ depletion occurred in 75.0% of the patients with CLL, and all patients with FL and DLBCL.

**Conclusions:**

The safety and efficacy of obinutuzumab monotherapy in Chinese patients with B-cell lymphomas were similar to that observed in previous studies in non-Chinese patients; no new safety signals were observed.

*Clinical trial registration ID* NCT01680991

**Electronic supplementary material:**

The online version of this article (10.1186/s40880-018-0300-5) contains supplementary material, which is available to authorized users.

## Background

B-cell lymphoproliferative disorders range from slow-growing indolent diseases, such as chronic lymphocytic leukemia (CLL) and follicular lymphoma (FL), to more aggressive diseases, such as diffuse large B-cell lymphoma (DLBCL). The incidence of CLL in the Chinese population is approximately one-tenth of that in Western countries [1]. In a nationwide collaborative study of 10,002 lymphoma patients by Li and colleagues in [2], DLBCL accounted for 50.2% of all cases of common B-cell lymphoma in China; FL and CLL/small lymphocytic leukemia accounted for 8.3% and 6.4%, respectively. Most B-cell lymphomas are characterized by the expression of CD20^+^, a membrane antigen implicated in cell-cycle initiation and differentiation [3]. Anti-CD20^+^ monoclonal antibodies, such as rituximab, have been shown to be effective for B-cell lymphomas when used in combination with chemotherapeutic agents [4–7]. However, many patients eventually relapse or become refractory upon repeated exposure. Thus, there is an ongoing need to develop more therapies that could effectively prolong remission and/or improve survival.

Obinutuzumab (GA101; GAZYVA/GAZYVARO) is a novel glycol-engineered type II anti-CD20^+^ monoclonal antibody, and has shown promising activity in phase Ib/II studies in patients with B-cell malignancies [8–11]. In stage II of the pivotal phase III CLL11 study in previously untreated CLL patients receiving background chlorambucil, obinutuzumab significantly prolonged progression-free survival (PFS) in comparison to rituximab (median PFS 29.2 vs. 15.4 months; hazard ratio 0.40; 95% confidence interval, 0.33–0.50, *P* <* 0.001*) [12, 13]. Based on results from the CLL11 study, obinutuzumab in combination with chlorambucil has been approved for the treatment of patients with previously untreated CLL in more than 60 countries [14, 15]. Obinutuzumab has also shown activity in patients with relapsed CD20^+^ indolent non-Hodgkin lymphoma (NHL). In the phase II GAUSS study in relapsed CD20^+^ indolent NHL patients, obinutuzumab produced a higher overall response rate than rituximab (44.6% vs. 33.3%; *P* = *0.08*) [16]. The efficacy and safety of obinutuzumab has also been assessed in the phase III GADOLIN study in patients with rituximab-refractory indolent NHL [17]. In this study, obinutuzumab plus bendamustine was associated with a 45% reduction in the risk of disease progression or death (assessed by an independent review committee) vs. bendamustine induction alone. Based on the results from GADOLIN, obinutuzumab has been approved for rituximab-refractory FL in the US and Europe [14, 15]. In all three trials, the adverse events (AEs) related to obinutuzumab alone, or in combination with other chemotherapeutic agents, were generally manageable.

To date, no ethnic sensitivity associated with the use of obinutuzumab has been reported. We have completed a trial (GERSHWIN; trial registration ID: NCT01680991) to evaluate obinutuzumab in Chinese patients with CD20^+^ relapsed/refractory malignant B-cell lymphomas. The primary outcome measure of pharmacokinetic profile of obinutuzumab following repeated intravenous dosing has been previously reported [18]. Here, we report the results of secondary outcome measures: safety, tolerability, efficacy, and pharmacodynamics.

## Patients and methods

### Study design and treatment

GERSHWIN was an open-label, multicenter, single-arm, phase Ib study, conducted in four sites in China between September 6, 2012 and August 15, 2013. The study was conducted in accordance with the Declaration of Helsinki and the International Conference on Harmonization guidelines for Good Clinical Practice. Approval from Institutional Review Boards and/or Independent Ethics Committees was obtained before the trial started.

Patients received a maximum of eight treatment cycles (24 weeks), each lasting for 21 days. On day 1 of each cycle, obinutuzumab was given intravenously at a dose of 1000 mg. In cycle 1, additional doses of obinutuzumab were administered on days 8 and 15. In patients with CLL, the first infusion of obinutuzumab on cycle 1 was given as a split dose over 2 days (100 mg at a rate of 25 mg/h over 4 h on day 1; 900 mg starting at 50 mg/h on day 2, increasing by 50 mg/h every 30 min, to a maximum of 400 mg/h) to minimize the risk of infusion-related reactions (IRRs). For the first infusion in patients with NHL, obinutuzumab was administered at an initial rate of 50 mg/h, and increased by 50 mg/h every 30 min to a maximum of 400 mg/h if there were no signs of IRRs. If the first obinutuzumab infusion was well tolerated by all study subjects, subsequent infusions were initiated at 100 mg/h and increased by 100 mg/h increments every 30 min to a maximum of 400 mg/h.

### Study population

Adult patients (> 18 years of age) with histologically documented CD20^+^ relapsed/refractory CLL, DLBCL, or FL; an Eastern Cooperative Oncology Group performance status of 0 or 1; and a life expectancy of > 6 months were eligible. With the exception of CLL, all patients had at least one bidimensionally measurable lesion (> 1.5 cm in its largest dimension by computed tomography). For patients with CLL, circulating lymphocyte cell assessments were an acceptable method of measurement.

To be eligible, patients with DLBCL or FL must have relapsed after, or had been refractory to, at least one standard chemotherapy, with or without rituximab. CLL patients must have relapsed after, or had been refractory to at least one chemotherapy regimen. Relapse was defined as disease recurrence after any documented history of response [complete response (CR), CR with incomplete bone marrow recovery (CRi; CLL patients only) or partial response (PR)] that lasted ≥ 6 months. Refractoriness was defined as progression or stable disease (SD) on treatment, or any response that was followed by progression < 6 months after treatment. All patients provided written informed consent to participate in the study.

Key exclusion criteria included the following: use of any investigational monoclonal antibody therapy within 6 months before the trial started; use of any anticancer vaccine; prior treatment with rituximab or radioimmunotherapy within 3 months of study entry; history of severe anaphylactic reactions to humanized or murine monoclonal antibodies; central nervous system lymphoma; and history of other malignancy or evidence of significant, uncontrolled concomitant diseases (including cardiovascular and pulmonary diseases). Full inclusion and exclusion criteria are shown in Additional file 1.

### Study endpoints and procedures

#### Safety and tolerability

Safety assessments included AEs, serious AEs (SAEs) and AEs of special interest [AESI; e.g., IRRs, serious neutropenia, infections, and tumor lysis syndrome (TLS)]. AEs were summarized using the Medical Dictionary for Regulatory Activities system organ class, and graded using the National Cancer Institute Common Terminology Criteria for Adverse Events, version 4.0. Safety analyses included laboratory assessments (hematology, biochemistry, and urinalysis), vital signs, and electrocardiograms (ECGs); tests for immunogenicity [human antichimeric antibodies (HACA) and human antihuman antibodies (HAHA)] were also performed.

#### Efficacy

Treatment response was assessed according to the International Workshop to Standardize Response criteria for NHL [19], and 2008 Guidelines of the International Workshop on CLL [20]. End-of-treatment (EoT) response was assessed 1 month after the cycle 8, day 1 infusion, or for treatment withdrawals after cycle 4. For patients with CLL, a further confirmation of response was performed 2 months after EoT. Best overall response was assessed at any time point during the study, prior to new anti-lymphoma/leukemia therapy. For both EoT and best overall response, patients were regarded as responders if they demonstrated CR, CR unconfirmed (CRu), CRi (CLL patients only), or PR. Patients were considered to be non-responders if they demonstrated SD or progressive disease (PD), or were missing response assessments. Follow-up was carried out up to 1 year after the last dose.

#### Pharmacodynamics

CD19^+^ depletion was defined as CD19^+^ cell count < 0.07 × 10^9^/L; recovery was defined as ≥ 0.07 × 10^9^/L. The duration of depletion was defined as the time between the first assessment of depletion and the first assessment when CD19^+^ B-cell count returned to at least the depletion level from baseline and was not followed by any further depletion time to recovery was defined as the time between the beginning of depletion and the first value after EoT that was ≥ 0.07 × 10^9^/L, not followed exclusively by depleted values.

### Analytical plan

Only descriptive analyses were performed; no formal hypothesis was tested.

## Results

### Patients

**Fig. 1:**
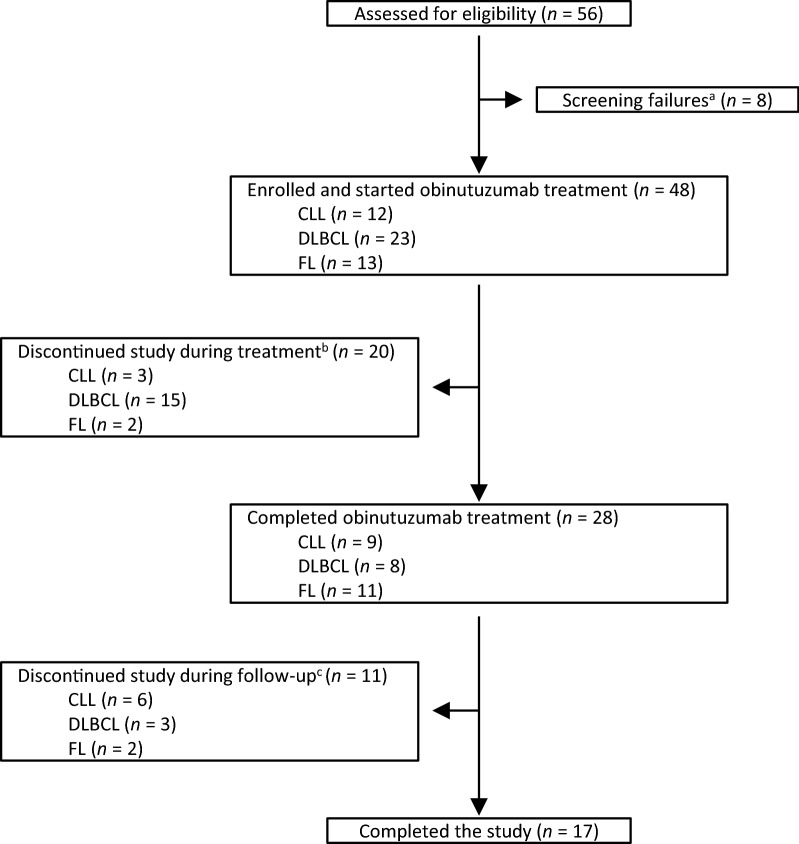
Patient flow through the study. ^a^Reasons for screening failures: three patients tested positive for Hepatitis B core antibody, two patients did not meet inclusion criteria number 1 (histologically documented CD20^+^ malignant disease), one patient did not meet inclusion criteria number 2 (relapsed/refractory CLL, FL, or DLBCL), one patient had positive hepatitis serology, and one patient had a hemoglobin value of 56 g/L. ^b^Reasons for discontinuation during treatment: AE (pneumonia), *n* = 1; PD, *n* = 9; protocol violation, *n* = 1; withdrawal by the patient, *n* = 3; physician decision, *n* = 6. ^c^Reasons for discontinuation during follow-up: death, *n* = 2; withdrawal by the patient, *n* = 4; PD, *n* = 1; physician decision, *n* = 2; other, *n* = 2 (patient could not visit due to poor physical condition, progression of disease confirmed in another hospital). *CLL* chronic lymphocytic leukemia, *DLBCL* diffuse large B-cell lymphoma, *FL* follicular lymphoma

A total of 56 patients were screened for eligibility, among which 48 were enrolled (Fig. 1): 12 (25.0%) with CLL, 23 (47.9%) with DLBCL, and 13 (27.1%) with FL. Most patients had advanced disease [Binet B or C (CLL); Ann Arbor III or IV (NHL)] (Table 1). The median number of previous anticancer treatments was 2 or 3 depending on disease type. Twenty-eight subjects (58.3%; 28/48) completed the planned treatment. Obinutuzumab was discontinued in 17 (35.4%) patients during cycles 1–4 and three (6.3%) patients during cycles 5–8.

**Table 1:** Demographics and disease characteristics of the subject who received at least one dose of obinutuzumab

Variable	CLL (*n* = 12)	DLBCL (*n* = 23)	FL (*n* = 13)	Overall (*n* = 48)
Age at baseline (years)				
Mean (sd)	60.7 (12.0)	53.3 (15.8)	55.1 (8.8)	55.6 (13.4)
Gender [*n* (%)]				
Male	7 (58.3)	11 (47.8)	8 (61.5)	26 (54.2)
Female	5 (41.7)	12 (52.2)	5 (38.5)	22 (45.8)
Weight (kg)				
Mean (sd)	60.83 (11.44)	62.33 (9.86)	64.27 (12.49)	62.48 (10.85)
Height (cm)				
Mean (sd)	161.0 (5.0)	164.1 (7.1)	166.3 (10.7)	163.9 (7.9)
ECOG at baseline [*n* (%)]				
0	2 (16.7)	8 (34.8)	8 (61.5)	18 (37.5)
1	10 (83.3)	15 (65.2)	5 (38.5)	30 (62.5)
Ann Arbor stage at diagnosis^a^ [*n* (%)]				
I	N/A	0	2 (15.4)	2 (5.6)
II	N/A	4 (17.4)	0	4 (11.1)
III	N/A	8 (34.8)	5 (38.5)	13 (36.1)
IV	N/A	7 (30.4)	3 (23.1)	10 (27.8)
Missing	N/A	4 (17.4)	3 (23.1)	7 (19.4)
Binet stage^a^ [*n* (%)]				
Stage A	1 (8.3)	N/A	N/A	1 (8.3)
Stage B	6 (50.0)	N/A	N/A	6 (50.0)
Stage C	2 (16.7)	N/A	N/A	2 (16.7)
Unknown	3 (25.0)	N/A	N/A	3 (25.0)
Number of previous lines of treatment				
Median	2.0	2.0	3.0	2.0
Minimum–maximum	1–7	1–11	1–6	1–11
Best response of prior treatment [*n* (%)]				
CR	1 (8.3)	9 (39.1)	2 (15.4)	12 (25.0)
PR	8 (66.7)	9 (39.1)	7 (53.8)	24 (50.0)
SD	1 (8.3)	2 (8.7)	0	3 (6.3)
PD	0	2 (8.7)	0	2 (4.2)
Missing	2 (16.7)	1 (4.3)	4 (30.8)	7 (14.6)
Duration of best response				
*n*	6	18	7	31
Mean (sd) (days)	355.2 (604.2)	152.5 (119.2)	159.4 (136.4)	193.3 (281.3)

*CLL* chronic lymphocytic leukemia, *CR* complete response, *DLBCL* diffuse large B-cell lymphoma, *ECOG* Eastern Cooperative Oncology Group, *FL* follicular lymphoma, *N/A* not applicable, *PR* partial response, *SD* stable disease, *sd* standard deviation

^a^ Patients were assessed according to Ann Arbor staging criteria for non-Hodgkin lymphoma and Binet staging criteria for CLL

### Safety and tolerability

All 48 enrolled patients received at least one dose of obinutuzumab and were included in the safety analysis. Median treatment duration was 20.8 weeks, with a median cumulative dose of 10,000 mg (Additional file 2). A total of 141 AEs were reported, mostly grade I–II (116/141, 82.3%). The number of patients who experienced at least one AE was 35 (CLL: 10/12, 83.3%; DLBCL: 18/23, 78.3%; FL: 7/13, 53.8%). The most common AE was IRR (CLL: 7/12, 58.3%; DLBCL: 5/23, 21.7%; FL: 3/13, 23.1%), with the highest rate in CLL patients (Additional file 3). Other common AEs included pyrexia (CLL: 6/12, 50.0%; DLBCL: 3/23, 13.0%; FL: 2/13, 15.4%) and cough (CLL: 4/12, 33.3%; DLBCL: 1/23, 4.3%; FL: 0). No patients withdrew from treatment as a result of treatment-related AEs.

Twenty-five grade III–V AEs were reported, with a seemingly higher rate in the CLL subgroup (CLL: 7/12, 58.3%; DLBCL: 5/23, 21.7%; FL: 3/13, 23.1%; Additional file 4). Infections and infestations were the most common grade III–V AEs across all three subgroups (CLL: 3/12, 25.0%; DLBCL: 2/23, 8.7%; FL: 2/13, 15.4%) (Additional file 4), with pneumonia accounting for two of the three events in the CLL subgroup. Grade III–V thrombocytopenia occurred in 16.7% (2/12) of the CLL patients. Neutropenia occurred in 15.4% (2/13) of the FL patients.

Thirteen SAEs were reported in nine patients, of which five were infections and infestations (CLL: 3/12, 25.0%; DLBCL: 1/23, 4.3%; FL: 1/13, 7.7%; Additional file 5). One SAE (grade III, pneumonia) led to the withdrawal of treatment in a patient with CLL, but was considered unrelated to treatment with obinutuzumab. In this case, pneumonia started on day 52, treatment was discontinued on day 66, and the patient was withdrawn from the study on day 90 (Table 2). The total rate of SAEs appeared higher in the CLL subgroup (Additional file 5). Six (6/48; 12.5%) patients reported a total of eight AESIs, including serious IRR (CLL: 1/12, 8.3%), serious neutropenia (CLL: 1/12, 8.3%; FL: 2/13, 15.4%), and serious infections (CLL: 3/12, 25.0%; DLBCL: 1/23, 4.3%; FL: 1/13, 7.7%). The patient with a serious IRR also developed a serious infection (pneumonia). No cases of TLS were reported. Two deaths occurred during the follow-up period: one in the CLL subgroup [interstitial lung disease (interstitial pneumonia; no testing for *Pneumocystis jiroveci* was performed), which occurred 60 days after the last treatment dose] was considered treatment-related; the other after disease progression in the FL group was considered unrelated to treatment.

**Table 2:** Adverse events

Variable	CLL (*n* = 12)	DLBCL (*n* = 23)	FL (*n* = 13)	Overall (*n* = 48)
Number of patients with at least one AE	10 (83.3)	18 (78.3)	7 (53.8)	35 (72.9)
Number of AEs	71	45	25	141
Number of deaths	1 (8.3)	0	1 (7.7)	2 (4.2)
Number of patients withdrawn from study due to an AE	1 (8.3)	0	0	1 (2.1)
Number of patients with at least one				
AE with fatal outcome	1 (8.3)	0	0	1 (2.1)
SAE	5 (41.7)	3 (13.0)	1 (7.7)	9 (18.8)
SAE leading to withdrawal from treatment	1 (8.3)	0	0	1 (2.1%)
Related SAE	4 (33.3)	1 (4.3)	1 (7.7)	6 (12.5)
AE leading to withdrawal from treatment	1 (8.3)	0	0	1 (2.1)
AE leading to treatment interruption	4 (33.3)	3 (13.0)	2 (15.4)	9 (18.8)
Related AE	9 (75.0)	12 (52.2)	7 (53.8)	28 (58.3)
Related AE leading to treatment interruption	4 (33.3)	3 (13.0)	2 (15.4)	9 (18.8)
Grade III–V AE (at greatest intensity)	7 (58.3)	5 (21.7)	3 (23.1)	15 (31.3)

Percentages are based on *n* in the column headings. Multiple occurrences of the same AE in one individual are counted only once except for the ‘total number of AEs’ row, in which multiple occurrences of the same AE are counted separately

Data are shown as n (%)

*AE* adverse event, *CLL* chronic lymphocytic leukemia, *DLBCL* diffuse large B-cell lymphoma, *FL* follicular lymphoma, *SAE* serious adverse event

There were no clinically significant abnormal laboratory test results, ECGs, or vital signs during the treatment period. Three clinically significant abnormal ECGs were recorded prior to dosing. On cycle 1, day 1, two patients with CLL and one with DLBCL had detectable HACA. At the 6-month follow-up visit, one with FL had detectable HAHA.

No evidence of hepatitis B virus (HBV) reactivation was observed in any subject. Patients with CLL who were positive for hepatitis B core antibodies (anti-HBc) and negative for hepatitis B surface antigen were eligible for participation if HBV DNA was undetectable, but were required to undergo monthly HBV DNA testing during the study period. Among the four patients with positive anti-HBc at screening, one patient refused to undergo monthly HBV DNA testing and withdrew from the study, one patient withdrew before the protocol requirement for HBV DNA testing was implemented, and the remaining two patients underwent monthly testing with no indication of HBV reactivation.

### Efficacy

The efficacy dataset included all 48 patients. EoT and best overall response rate were higher in the CLL and FL subgroups than in the DLBCL subgroup (Table 3). In the CLL subgroup, the PR rate at EoT and best overall response rate were 58.3% (7/12) and 75.0% (9/12), respectively. In the FL subgroup, PR rate at EoT and best overall response rate were 46.2% (6/13) and 61.5% (8/13), respectively. In the DLBCL subgroup, EoT and best overall response rate were 13.0% (1/23 CRu; 2/23 PR) and 26.1% (1/23 CRu; 5/23 PR), respectively. No CR was reported in any patient.

**Table 3:** End of treatment and best overall response rate

Variable	CLL (*n* = 12)		DLBCL (*n* = 23)		FL (*n* = 13)		Overall (*n* = 48)	
	EoT response	Best overall response	EoT response	Best overall response	EoT response	Best overall response	EoT response	Best overall response
Responders, *n* (%)	7 (58.3)	9 (75.0)	3 (13.0)	6 (26.1)	6 (46.2)	8 (61.5)	16 (33.3)	23 (47.9)
Non-responders, *n* (%)	5 (41.7)	3 (25.0)	20 (87.0)	17 (73.9)	7 (53.8)	5 (38.5)	32 (66.7)	25 (52.1)
95% CI for response rate	27.67–84.83	42.81–94.51	2.78–33.59	10.23–48.41	19.22–74.87	31.58–86.14	20.40–48.41	33.29–62.81
CR, *n*	0	0	0	0	0	0	0	0
95% CI	0.00–26.46	0.00–26.46	0.00–14.82	0.00–14.82	0.00–24.71	0.00–24.71	0.00–7.40	0.00–7.40
CRi, *n*	0	0	0	0	0	0	0	0
95% CI	0.00–26.46	0.00–26.46	0.00–14.82	0.00–14.82	0.00–24.71	0.00–24.71	0.00–7.40	0.00–7.40
CRu, *n* (%)	0	0	1 (4.3)	1 (4.3)	0	0	1 (2.1)	1 (2.1)
95% CI	0.00–26.46	0.00–26.46	0.11–21.95	0.11–21.95	0.00–24.71	0.00–24.71	0.05–11.07	0.05–11.07
PR, *n* (%)	7 (58.3)	9 (75.0)	2 (8.7)	5 (21.7)	6 (46.2)	8 (61.5)	15 (31.3)	22 (45.8)
95% CI	27.67–84.83	42.81–94.51	1.07–28.04	7.46–43.70	19.22–74.87	31.58–86.14	18.66–46.25	31.37–60.83
SD, *n* (%)	1 (8.3)	1 (8.3)	2 (8.7)	6 (26.1)	4 (30.8)	4 (30.8)	7 (14.6)	11 (22.9)
95% CI	0.21–38.48	0.21–38.48	1.07–28.04	10.23–48.41	9.09–61.43	9.09–61.43	6.07–27.76	12.03–37.31
PD, *n* (%)	2 (16.7)	0	15 (65.2)	9 (39.1)	3 (23.1)	1 (7.7)	20 (41.7)	10 (20.8)
95% CI	2.09–48.41	0.00–26.46	42.73–83.62	19.71–61.46	5.04–53.81	0.19–36.03	27.61–56.79	10.47–34.99
Missing or non-evaluable, *n* (%)	2 (16.7)	2 (16.7)	3 (13.0)	2 (8.7)	0	0	5 (10.4)	4 (8.3)

95% CI for rates were constructed using Clopper–Pearson method. Patients were classified as missing or non-evaluable if no post-baseline response assessments were available or all post-baseline response baseline assessments were un-evaluable

*CLL* chronic lymphocytic leukemia, *CI* confidence interval, *CR* complete response, *CRi* CR with incomplete bone marrow recovery (CRi; CLL patients only), *CRu* CR unconfirmed, *DLBCL* diffuse large B-cell lymphoma, *EoT* end of treatment, *FL* follicular lymphoma, *PR* partial response, *SD* stable disease

### Pharmacodynamics

CD19^+^ B-cell depletion (count < 0.07 × 10^9^/L) occurred in 75.0% (9/12) with CLL, 100% (13/13) of the patients with FL, and 100% (23/23) with DLBCL. The FL subgroup had the longest median duration of depletion, 465 days, compared with 244 days for CLL and 92 days for DLBCL. Subsequently, 33.3% (4/12) of patients with CLL, 7.7% (1/13) with FL, and 4.3% (1/23) with DLBCL experienced B-cell recovery (CD19^+^ B-cell count ≥ 0.07 × 10^9^/L). One patient with CLL and one with FL who experienced B-cell recovery had PD: time to recovery was 419 days (CLL) and 331 days (FL), respectively. For the three patients with CLL who did not have PD, the median time to recovery was 182 days, and for DLBCL (one patient only), it was 515 days.

## Discussion

GERSHWIN showed that obinutuzumab monotherapy has acceptable safety and tolerability profiles in Chinese patients with relapsed/refractory B-cell lymphomas; no new safety signals were observed. Obinutuzumab also showed encouraging efficacy, particularly in subjects with CLL.

Consistent with previous reports in Caucasian populations [21], IRRs, pyrexia, and cough were the most common AEs in all three disease types in the current study. All IRRs occurred during or within 24 h after the end of the obinutuzumab infusion; all were grade I or II, and manageable. The rate of IRRs appeared to be highest in patients with CLL (58.3%). Recent findings by Illidge et al. in [21] and Freeman et al. in [22] suggest that CLL patients with higher CD20^+^ expression are at greater risk of developing severe IRRs to obinutuzumab. The IRR rate observed in the current study was lower than reported by the GAUGUIN study despite similar intervention (monotherapy) and patient characteristics (relapsed/refractory B-cell lymphomas). In GAUGUIN, the rate of IRR in patients with relapsed/refractory CD20^+^ CLL was 100% (13/13 and 20/20 in phase I and II, respectively) [9]. In patients with relapsed/refractory CD20^+^ DLBCL/mantle cell lymphoma and indolent NHL in the GAUGUIN study, the IRR rate was 75.0% and 72.5%, respectively, and the majority were grade I or II and manageable [10, 11]. The IRR rate in our study was also lower than that reported in a phase I dose-finding study of obinutuzumab in 12 Japanese patients with relapsed/refractory CD20^+^ NHL, in which all patients experienced an IRR [23]. Awareness of recommended measures to prevent the development of IRR and other AEs during obinutuzumab therapy gained by some of the investigators in the current study during previous clinical trials may have contributed the lower IRR rate in the GERSHWIN study. However, it is important to note that such comparisons are limited by the differences in patient numbers and design between these studies. No TLS was reported in the current study. Previous studies in Caucasian patients, including CLL11 [12] and GAUGUIN [9, 11], reported neutropenia as a major AE after obinutuzumab treatment. In the current study, grade III–V neutropenia was reported in three cases, but resolved spontaneously or with treatment; none required dose adjustment/discontinuation. One patient with CLL died of interstitial lung disease on day 252 (82 days after the last dose of obinutuzumab); the infection started 60 days after the last dose and was considered treatment-related.

Obinutuzumab efficacy in the current study was similar to that seen in non-Chinese patients in previous studies (e.g., GAUGUIN and the phase I Japanese dose-finding study). The EoT response rate in the overall analysis was 33.3% (CLL: 58.3%; DLBCL: 13.0%; FL: 46.2%) and the best overall response rate was 47.9% (CLL: 75.0%; DLBCL: 26.1%; FL: 61.5%). Most patients with CLL achieved a PR at EoT (7/12; 58.3%). In GAUGUIN phase II (of relapsed/refractory CD20^+^ indolent NHL patients; 85.0% had FL), overall response rates ranged from 21.4% to 50.0% in the FL subgroup (dependent on obinutuzumab dosage), and best overall response rate ranged from 35.7% to 60.0% [11]. In the phase I Japanese dose-finding study (66.7% of patients had FL), the EoT response rate was 58.3% [23]. By comparison, in GAUGUIN phase II (in patients with relapsed/refractory CD20^+^ DLBCL and mantle cell lymphoma, 62.5% DLBCL), EoT and best overall response rate in the DLBCL subgroup were 28.0% and 32.0%, respectively [10].

Limitations of the current study include the small sample size (*n *= 48) and lack of a control arm. Nevertheless, the results are encouraging and are generally comparable with previous studies in non-Chinese patients.

## Conclusions

The safety profile and efficacy of obinutuzumab monotherapy in Chinese patients with CD20^+^ malignant B-cell lymphomas was similar to that observed in previous studies in non-Chinese patient populations. No new safety signals were observed.

## Additional files

**Additional file 1.** Detailed inclusion and exclusion criteria.

**Additional file 2.** Study drug exposure.

**Additional file 3. ** Incidence of AEs of any grade reported in ≥10% of patients (in any patient population).

**Additional file 4.** Summary of patients with AEs NCI-CTCAE grade III–V.

**Additional file 5.** Summary of SAEs.

## Abbreviations

AE: adverse event
AESI: AEs of special interest
CLL: chronic lymphocytic leukemia
CR: complete response
CRi: CR with incomplete bone marrow recovery
CRu: CR unconfirmed
DLBCL: diffuse large B-cell lymphoma
ECGs: electrocardiograms
EoT: end-of-treatment
FL: follicular lymphoma
HACA: human antichimeric antibodies
HAHA: human antihuman antibodies
IRRs: infusion-related reactions
NHL: non-Hodgkin lymphoma
PFS: progression-free survival
PR: partial response
SAEs: serious AEs
SD: stable disease
TLS: tumor lysis syndrome
PD: progressive disease
