# Supplementary material for: Safety and efficacy of obinutuzumab in Chinese patients with B-cell lymphomas: a secondary analysis of the GERSHWIN trial
Source: Cancer Commun (Lond). 2018 May 30;38:31. doi: 10.1186/s40880-018-0300-5 (PMC5993131; doi:10.1186/s40880-018-0300-5)
Supplement: Supplementary file 1 — Additional file 1. Detailed inclusion and exclusion criteria. [file 40880_2018_300_MOESM1_ESM.docx]

**Additional file 1**

**Inclusion criteria**

To be eligible, subjects must meet all following criteria:

1. Histologically documented CD20^+^ malignant disease (B-cell lymphoma or B- chronic lymphocytic leukemia [CLL; B-CLL confirmed by NCI Working Group Criteria, 2008]).
2. Relapsed or refractory follicular lymphoma (FL), diffuse large B-cell lymphoma (DLBCL) or CLL:

a) Patients with FL or DLBCL could be included if they had failed (relapsing after or refractory to) at least one standard chemotherapy (recommended by the Chinese guideline or judged to be appropriate by the investigator) with or without rituximab at any point in their treatment history;

b) Patients with CLL could be included if they had relapsed or were refractory to at least one chemotherapy regimen at any point in their treatment history;

*Relapse and refractoriness were defined as follows:*

- *Relapse was defined as disease recurrence after any documented history of response (complete response [CR], CR with incomplete bone marrow recovery [CRi (CLL only)] or partial response [PR]) of ≥6 months;*
- *Refractoriness was defined as progression on treatment or stable disease (SD), or any response that was followed by progression* <*6 months after treatment.*

3. Patients with FL and DLBCL had to have at least one bi-dimensionally measurable lesion (>1.5 cm in its largest dimension by computed tomography scan). For CLL patients, circulating lymphocyte cell assessments were an acceptable method of measurement. Note that all measurable and evaluable disease must have been assessed and documented prior to the initiation of obinutuzumab treatment. Tumor response was assessed according to the International Workshop to standardize response criteria for non-Hodgkin lymphoma [19], and the 2008 guidelines of the International Workshop on CLL [20].

4. Able and willing to provide written informed consent and to comply with the study protocol.

5. Age >18 years.

6. Eastern Cooperative Oncology Group performance status of 0 or 1.

7. Life expectancy >6 months.

**Exclusion criteria**

Subjects were excluded upon any of the following conditions:

1. Prior use of any investigational monoclonal antibody therapy within 6 months of study start.
2. Prior use of any anticancer vaccine.
3. Prior administration of rituximab within 3 months of study entry.
4. Prior administration of radioimmunotherapy within 3 months of study entry.
5. History of severe allergic or anaphylactic reactions to humanized or murine monoclonal antibodies. Known sensitivity or allergy to murine products.
6. Central nervous system lymphoma.
7. History of other malignancy that could affect compliance with the protocol or interpretation of results. Patients with a history of curatively treated basal or squamous cell carcinoma of the skin or *in situ* carcinoma of the cervix were generally eligible, provided the tumor was treated with curative intent at least 2 years prior to study entry.
8. Evidence of significant, uncontrolled concomitant diseases that could affect compliance with the protocol or interpretation of results, including significant cardiovascular disease (such as New York Heart Association Class III or IV cardiac disease, myocardial infarction within the last 6 months, unstable arrhythmias, or unstable angina) or pulmonary disease (including obstructive pulmonary disease and history of symptomatic bronchospasm).
9. Known active bacterial, viral (including human immunodeficiency virus [HIV]), fungal, mycobacterial, or other infection (excluding fungal infections of nail beds), or any major episode of infection requiring hospitalization or treatment with intravenous antibiotics (for intravenous antibiotics this pertains to completion of last course of antibiotic treatment) within 4 weeks of receiving the first dose of obinutuzumab.
10. Recent major surgery (within 4 weeks prior to receiving first dose of obinutuzumab), other than for diagnosis.
11. Any of the following abnormal laboratory values:

- Creatinine clearance of ≤40 mL/min, calculated according to the Cockcroft-Gault formula
- Aspartate aminotransferase or alanine aminotransferase >2.5 × upper limit of normal (ULN) for >2 weeks; bilirubin >3 × ULN unless due to underlying disease
- Platelet count <75 × 10^9^/L
- Neutrophils <1.5 × 10^9^/L
- Hemoglobin <8 g/dL
- Prothrombin time (PT)/international normalized ratio (INR) >2 × ULN if not on therapeutic/prophylactic anticoagulation.

**NOTE:** Patients with cell counts below the thresholds listed above could be considered eligible if, in the investigator’s opinion, this was due to bone marrow infiltration.

1. Positive test result for HIV.
2. History of confirmed progressive multifocal leukoencephalopathy.
3. Positive hepatitis serology:

Hepatitis B (HBV): Patients with positive serology for hepatitis B, defined as positive for hepatitis B surface antigen (HBsAg) or hepatitis B core antibody (anti-HBc). Patients with CLL who were positive for anti-HBc and negative for HbsAg could be included if HBV DNA was undetectable as confirmed by a central lab. These patients had to be willing to undergo monthly DNA testing.

Hepatitis C (HCV): Patients with positive hepatitis C serology unless HCV (RNA) was confirmed negative.

1. Women who were pregnant or lactating.
2. Fertile men or women of childbearing potential unless: (1) surgically sterile or ≥2 years after the onset of menopause, (2) willing to use a highly effective contraceptive method such as oral contraceptives, intrauterine device, sexual abstinence or barrier method of contraception in conjunction with spermicidal jelly during study treatment and in female patients for 12 months (male patients for 3 months) after end of obinutuzumab treatment.
3. Treatment within a clinical study within 30 days prior to study entry.
4. Vaccination with a live vaccine within 28 days prior to the first dose.
