# Supplementary material for: Safety and efficacy of obinutuzumab in Chinese patients with B-cell lymphomas: a secondary analysis of the GERSHWIN trial
Source: Cancer Commun (Lond). 2018 May 30;38:31. doi: 10.1186/s40880-018-0300-5 (PMC5993131; doi:10.1186/s40880-018-0300-5)
Supplement: Supplementary file 2 — Additional file 2. Study drug exposure. [file 40880_2018_300_MOESM2_ESM.docx]

**Additional file 2.** Study drug exposure

|  | **CLL (*n* = 12)** | **DLBCL (*n* = 23)** | **FL (*n* = 13)** | **Overall (*n* = 48)** |
| --- | --- | --- | --- | --- |
| Treatment duration (weeks) | | | | |
| Mean (SD) | 17.7 (7.3) | 12.1 (7.9) | 19.2 (4.5) | 15.4 (7.6) |
| Median | 21.0 | 9.0 | 21.0 | 20.8 |
| Minimum–maximum | 0–24 | 0–21 | 9–22 | 0–24 |
| Number of cycles |  |  |  |  |
| Mean (SD) | 6.75 (2.38) | 5.00 (2.68) | 7.38 (1.50) | 6.08 (2.53) |
| Median | 8.00 | 4.00 | 8.00 | 8.00 |
| Minimum–maximum | 1.0–8.0 | 1.0–8.0 | 4.0–8.0 | 1.0–8.0 |
| Total cumulative dose (mg) | | | | |
| Mean (SD) | 8,583.3 (2,843.1) | 6,913.0 (2,843.1) | 9,384.6 (1,502.1) | 8,000.0 (2,728.9) |
| Median | 10,000.0 | 6,000.0 | 10,000.0 | 10,000.0 |
| Minimum–maximum | 1,000–10,000 | 1,000–10,000 | 6,000–10,000 | 1,000–10,000 |

CLL, chronic lymphocytic leukemia; DLBCL, diffuse large B-cell lymphoma; FL, follicular lymphoma; SD, standard deviation.

Treatment duration is the date of the last dose of study medication minus the date of the first dose,
plus 1 day.
