# Supplementary material for: Safety and efficacy of obinutuzumab in Chinese patients with B-cell lymphomas: a secondary analysis of the GERSHWIN trial
Source: Cancer Commun (Lond). 2018 May 30;38:31. doi: 10.1186/s40880-018-0300-5 (PMC5993131; doi:10.1186/s40880-018-0300-5)
Supplement: Supplementary file 3 — Additional file 3. Incidence of AEs of any grade reported in ≥10% of patients (in any patient population). [file 40880_2018_300_MOESM3_ESM.docx]

**Additional file 3.** AEs of any grade reported in ≥10% patients (in any subgroup)

|  | **CLL (*n* = 12)** | **DLBCL (*n* = 23)** | **FL (*n* = 13)** | **Overall (*n* = 48)** |
| --- | --- | --- | --- | --- |
| Number of patients with at least one AE, *n* (%) | 10 (83.3) | 18 (78.3) | 7 (53.8) | 35 (72.9) |
| Number of AEs, *n* | 71 | 45 | 25 | 141 |
| Injury, poisoning, and procedural complications | | | | |
| Number of patients with at least one AE, *n* (%) | 7 (58.3) | 5 (21.7) | 3 (23.1) | 15 (31.3) |
| Infusion-related reaction, *n* (%) | 7 (58.3) | 5 (21.7) | 3 (23.1) | 15 (31.3) |
| Number of AEs, *n* | 11 | 5 | 3 | 19 |
| General disorders and administration site conditions | | | | |
| Number of patients with at least one AE, *n* (%) | 6 (50.0) | 5 (21.7) | 3 (23.1) | 14 (29.2) |
| Pyrexia, *n* (%) | 6 (50.0) | 3 (13.0) | 2 (15.4) | 11 (22.9) |
| Chills, *n* (%) | 3 (25.0) | 1 (4.3) | 0 | 4 (8.3) |
| Number of AEs, *n* | 16 | 6 | 5 | 27 |
| Infections and infestations | | | | |
| Number of patients with at least one AE, *n* (%) | 5 (41.7) | 5 (21.7) | 4 (30.8) | 14 (29.2) |
| Pneumonia, *n* (%) | 2 (16.7) | 1 (4.3) | 1 (7.7) | 4 (8.3) |
| Number of AEs, *n* | 5 | 6 | 5 | 16 |
| Blood and lymphatic system disorders | | | | |
| Number of patients with at least one AE, *n* (%) | 7 (58.3) | 2 (8.7) | 2 (15.4) | 11 (22.9) |
| Thrombocytopenia, *n* (%) | 3 (25.0) | 1 (4.3) | 0 | 4 (8.3) |
| Anemia, *n* (%) | 2 (16.7) | 0 | 1 (7.7) | 3 (6.3) |
| Neutropenia, *n* (%) | 1 (8.3) | 0 | 2 (15.4) | 3 (6.3) |
| Number of AEs, *n* | 9 | 3 | 5 | 17 |

| Gastrointestinal disorders | | | | |
| --- | --- | --- | --- | --- |
| Number of patients with at least one AE, *n* (%) | 3 (25.0) | 4 (17.4) | 2 (15.4) | 9 (18.8) |
| Diarrhea, *n* (%) | 2 (16.7) | 0 | 1 (7.7) | 3 (6.3) |
| Number of AEs, *n* | 7 | 7 | 2 | 16 |
| Investigations | | | | |
| Number of patients with at least one AE, *n* (%) | 3 (25.0) | 5 (21.7) | 1 (7.7) | 9 (18.8) |
| ALT increased, *n* (%) | 0 | 3 (13.0) | 0 | 3 (6.3) |
| AST increased, *n* (%) | 0 | 3 (13.0) | 0 | 3 (6.3) |
| Number of AEs, *n* | 7 | 8 | 1 | 16 |
| Respiratory, thoracic, and mediastinal disorders | | | | |
| Number of patients with at least one AE, *n* (%) | 5 (41.7) | 1 (4.3) | 0 | 6 (12.5) |
| Cough, *n* (%) | 4 (33.3) | 1 (4.3) | 0 | 5 (10.4) |
| Number of AEs, *n* | 5 | 1 | 0 | 6 |
| Vascular disorders | | | | |
| Number of patients with at least one AE, *n* (%) | 2 (16.7) | 1 (4.3) | 1 (7.7) | 4 (8.3) |
| Hypertension, *n* (%) | 2 (16.7) | 1 (4.3) | 0 | 3 (6.3) |
| Number of AEs, *n* | 2 | 1 | 1 | 4 |

AEs, adverse events; ALT, alanine aminotransferase; AST, aspartate aminotransferase; CLL, chronic lymphocytic leukemia; DLBCL, diffuse large B-cell lymphoma; FL, follicular lymphoma.

Percentages are based on *n* in the column headings. For frequency counts by preferred term, multiple occurrences of the same AE in an individual were counted only once. For frequency counts of ‘number of AEs’ rows, multiple occurrences of the same AE in an individual are counted separately.
