# Supplementary material for: Safety and efficacy of obinutuzumab in Chinese patients with B-cell lymphomas: a secondary analysis of the GERSHWIN trial
Source: Cancer Commun (Lond). 2018 May 30;38:31. doi: 10.1186/s40880-018-0300-5 (PMC5993131; doi:10.1186/s40880-018-0300-5)
Supplement: Supplementary file 4 — Additional file 4. Summary of patients with AEs NCI-CTCAE grade III–V. [file 40880_2018_300_MOESM4_ESM.docx]

**Additional file 4.** Summary of NCI-CTCAE grade III–V AEs

|  | **CLL (*n* = 12)** | **DLBCL (*n* = 23)** | **FL (*n* = 13)** | **Overall (*n* = 48)** |
| --- | --- | --- | --- | --- |
| Number of patients with at least one AE, *n* (%) | 7 (58.3) | 5 (21.7) | 3 (23.1) | 15 (31.3) |
| Number of AEs, *n* | 13 | 6 | 6 | 25 |
| General disorders and administration site conditions | | | | |
| Number of patients with at least one AE, *n* (%) | 0 | 2 (8.7) | 0 | 2 (4.2) |
| Pain, *n* (%) | 0 | 1 (4.3) | 0 | 1 (2.1) |
| Submandibular mass, *n* (%) | 0 | 1 (4.3) | 0 | 1 (2.1) |
| Number of AEs, *n* | 0 | 2 | 0 | 2 |
| Infections and infestations | | | | |
| Number of patients with at least one AE, n (%) | 3 (25.0) | 2 (8.7) | 2 (15.4) | 7 (14.6) |
| Pneumonia, *n* (%) | 2 (16.7) | 0 | 1 (7.7) | 3 (6.3) |
| Urinary tract infection, *n* (%) | 1 (8.3) | 0 | 1 (7.7) | 2 (4.2) |
| Infected cyst, *n* (%) | 0 | 1 (4.3) | 0 | 1 (2.1) |
| Urethritis, *n* (%) | 0 | 1 (4.3) | 0 | 1 (2.1) |
| Number of AEs, *n* | 3 | 2 | 2 | 7 |
| Blood and lymphatic system disorders | | | | |
| Number of patients with at least one AE, *n* (%) | 5 (41.7) | 0 | 2 (15.4) | 7 (14.6) |
| Neutropenia, *n* (%) | 1 (8.3) | 0 | 2 (15.4) | 3 (6.3) |
| Anemia, *n* (%) | 1 (8.3) | 0 | 1 (7.7) | 2 (4.2) |
| Thrombocytopenia, *n* (%) | 2 (16.7) | 0 | 0 | 2 (4.2) |
| Leukocytosis, *n* (%) | 1 (8.3) | 0 | 0 | 1 (2.1) |
| Number of AEs, *n* | 5 | 0 | 4 | 9 |

| Gastrointestinal disorders | | | | |
| --- | --- | --- | --- | --- |
| Number of patients with at least one AE, *n* (%) | 1 (8.3) | 1 (4.3) | 0 | 2 (4.2) |
| Diarrhea, *n* (%) | 1 (8.3) | 0 | 0 | 1 (2.1) |
| Gastritis, *n* (%) | 0 | 1 (4.3) | 0 | 1 (2.1) |
| Number of AEs, *n* | 1 | 1 | 0 | 2 |
| Investigations | | | | |
| Number of patients with at least one AE, *n* (%) | 1 (8.3) | 1 (4.3) | 0 | 2 (4.2) |
| ALT increased, *n* (%) | 0 | 1 (4.3) | 0 | 1 (2.1) |
| Neutrophil count decreased,  *n* (%) | 1 (8.3) | 0 | 0 | 1 (2.1) |
| Platelet count decreased, *n* (%) | 1 (8.3) | 0 | 0 | 1 (2.1) |
| White blood cell count decreased, *n* (%) | 1 (8.3) | 0 | 0 | 1 (2.1) |
| Number of AEs, *n* | 3 | 1 | 0 | 4 |
| Respiratory, thoracic, and mediastinal disorders | | | | |
| Number of patients with at least one AE, *n* (%) | 1 (8.3) | 0 | 0 | 1 (2.1) |
| Interstitial lung disease, *n* (%) | 1 (8.3) | 0 | 0 | 1 (2.1) |
| Number of AEs, *n* | 1 | 0 | 0 | 1 |

AE, adverse event; ALT, alanine aminotransferase; AST, aspartate aminotransferase; CLL, chronic lymphocytic leukemia; DLBCL, diffuse large B-cell lymphoma; FL, follicular lymphoma; NCI-CTCAE, National Cancer Institute-Common Toxicity Criteria for AEs.

Percentages are based on *n* in the column headings. For frequency counts by preferred term, multiple occurrences of the same AE in an individual were counted only once. For frequency counts of ‘number of AEs’ rows, multiple occurrences of the same AE in an individual are counted separately.
