# Supplementary material for: Safety and efficacy of obinutuzumab in Chinese patients with B-cell lymphomas: a secondary analysis of the GERSHWIN trial
Source: Cancer Commun (Lond). 2018 May 30;38:31. doi: 10.1186/s40880-018-0300-5 (PMC5993131; doi:10.1186/s40880-018-0300-5)
Supplement: Supplementary file 5 — Additional file 5. Summary of SAEs. [file 40880_2018_300_MOESM5_ESM.docx]

**Additional file 5.** Summary of SAEs

|  | **CLL (*n* = 12)** | **DLBCL (*n* = 23)** | **FL (*n* = 13)** | **Overall (*n* = 48)** |
| --- | --- | --- | --- | --- |
| Number of patients with at least one SAE, *n* (%) | 5 (41.7) | 3 (13.0) | 1 (7.7) | 9 (18.8) |
| Number of SAEs, *n* | 7 | 3 | 3 | 13 |
| General disorders and administration site conditions | | | | |
| Number of patients with at least one SAE, *n* (%) | 0 | 1 (4.3) | 0 | 1 (2.1) |
| Submandibular mass, *n* (%) | 0 | 1 (4.3) | 0 | 1 (2.1) |
| Number of SAEs, *n* | 0 | 1 | 0 | 1 |
| Infections and infestations | | | | |
| Number of patients with at least one SAE, *n* (%) | 3 (25.0) | 1 (4.3) | 1 (7.7) | 5 (10.4) |
| Pneumonia, *n* (%) | 2 (16.7) | 0 | 1 (7.7) | 3 (6.3) |
| Urinary tract infection, *n* (%) | 1 (8.3) | 0 | 0 | 1 (2.1) |
| Infected cyst, *n* (%) | 0 | 1 (4.3) | 0 | 1 (2.1) |
| Number of SAEs, *n* | 3 | 1 | 1 | 5 |
| Blood and lymphatic system disorders | | | | |
| Number of patients with at least one SAE, *n* (%) | 2 (16.7) | 0 | 1 (7.7) | 3 (6.3) |
| Neutropenia, *n* (%) | 1 (8.3) | 0 | 1 (7.7) | 2 (4.2) |
| Thrombocytopenia, *n* (%) | 1 (8.3) | 0 | 0 | 1 (2.1) |
| Number of SAEs, *n* | 2 | 0 | 2 | 4 |
| Gastrointestinal disorders | | | | |
| Number of patients with at least one SAE, n (%) | 1 (8.3) | 0 | 0 | 1 (2.1) |
| Diarrhea, *n* (%) | 1 (8.3) | 0 | 0 | 1 (2.1) |
| Number of SAEs, *n* | 1 | 0 | 0 | 1 |

| Investigations | | | | |
| --- | --- | --- | --- | --- |
| Number of patients with at least one SAE, *n* (%) | 0 | 1 (4.3) | 0 | 1 (2.1) |
| Platelet count decreased, *n* (%) | 0 | 1 (4.3) | 0 | 1 (2.1) |
| Number of SAEs, *n* | 0 | 1 | 0 | 1 |
| Respiratory, thoracic, and mediastinal disorders | | | | |
| Number of patients with at least one SAE, *n* (%) | 1 (8.3) | 0 | 0 | 1 (2.1) |
| Interstitial lung disease, *n* (%) | 1 (8.3) | 0 | 0 | 1 (2.1) |
| Number of SAEs, *n* | 1 | 0 | 0 | 1 |

CLL, chronic lymphocytic leukemia; DLBCL, diffuse large B-cell lymphoma; FL, follicular lymphoma; SAE, serious adverse event.

Percentages are based on *n* in the column headings. For frequency counts by preferred term, multiple occurrences of the same SAE in an individual were counted only once. For frequency counts of ‘number of SAEs’ rows, multiple occurrences of the same SAE in an individual are counted separately.
